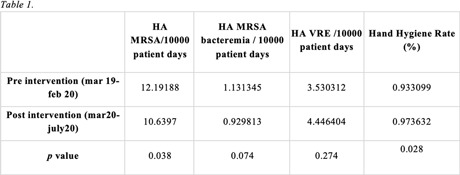# Discontinuation of Contact Precautions in Patients with Nosocomial MRSA and VRE Infections During the COVID-19 Pandemic

**DOI:** 10.1017/ash.2021.44

**Published:** 2021-07-29

**Authors:** Marisa Hudson, Mayar Al Mohajer

## Abstract

**Background:** Gaps exist in the evidence supporting the benefits of contact precautions for the prevention of methicillin-resistant *Staphylococcus aureus* (MRSA) and vancomycin-resistant enterococci (VRE). The Centers for Disease Control and Prevention allow suspending contact precautions for MRSA and VRE in cases of gown shortages, as we have seen during the COVID-19 pandemic. We evaluated the impact of discontinuing isolation precautions in hospitalized patients with MRSA and VRE infection, due to gown shortage, on the rate of hospital-acquired (HA) MRSA and VRE infections. **Methods:** A retrospective chart review was performed on adult patients (n = 2,200) with established MRSA or VRE infection at 5 hospitals in CommonSpirit Health, Texas Division, from March 2019 to October 2020. Data including demographics, infection site, documented symptoms, and antibiotic use were stratified based on patient location (floor vs ICU). Rates of hospital-acquired MRSA and VRE infection before and after the discontinuation of isolation (implemented in March 2020) were compared. Incidence density rate was used to assess differences in the rate of MRSA and VRE infections between pre- and postintervention groups. **Results:** The rate of hospital-acquired (HA) MRSA infection per 10,000 patient days before the intervention (March 19–February 20) was 12.19, compared to 10.64 after the intervention (March 20–July 20) (*P* = .038). The rates of HA MRSA bacteremia were 1.13 and 0.93 for the pre- and postintervention groups, respectively (*P* = .074). The rates of HA VRE per 10,000 patient days were 3.53 and 4.44 for the pre- and postintervention groups, respectively (*P* = .274). The hand hygiene rates were 0.93 before the intervention and 0.97 after the intervention (*P* = .028). **Conclusions:** Discontinuing isolation from MRSA and VRE in the hospital setting did not lead to a statistically significant increase in hospital-acquired MRSA or VRE infections. In fact, rates of hospital-acquired MRSA decreased, likely secondary to improvements in hand hygiene during this period. These results support the implementation of policies for discontinuing contact isolation for hospitalized patients with documented MRSA or VRE infection, particularly during shortages of gowns.

**Funding:** No

**Disclosures:** None

**Figure 1.**
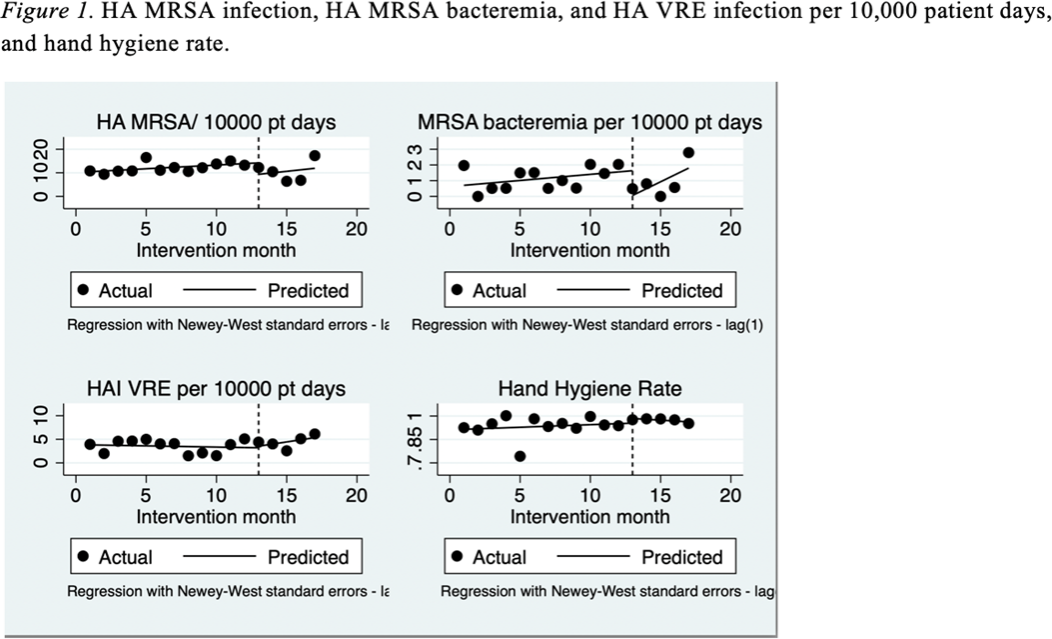

**Table 1.**